# Knowledge, attitudes, and practices of cataract patients in Shenzhen regarding cataract treatment

**DOI:** 10.3389/fpubh.2025.1697694

**Published:** 2026-01-21

**Authors:** Yuanjiao Qiao, Biyun Liang, Qiang Li, Dongyue Liu, Zengzhi Wang, Yanli Wang, Lishi Luo, Xiaosheng Huang, Wenqun Xi, Xinhua Liu, Kun Zeng

**Affiliations:** 1 Department of Cataract, Shenzhen Eye Hospital, Shenzhen Eye Medical Center, Southern Medical University, Shenzhen, Guangdong, China; 2 Department of Ophthalmology, Shenzhen People's Hospital, The Second Clinical Medical College, Jinan University, Shenzhen, Guangdong, China; 3 The First Affiliated Hospital, Southern University of Science and Technology, Shenzhen, Guangdong, China; 4 The Second Clinical Medical College, Jinan University, Shenzhen, Guangdong, China; 5 Department of Ophthalmology, Nanshan District People's Hospital, Shenzhen, Guangdong, China

**Keywords:** cataract, cross-sectional study, health behavior, health knowledge, attitudes, practice, patient education, survey and questionnaire

## Abstract

**Purpose:**

This study aimed to explore knowledge, attitudes, and practices (KAP) regarding cataracts among patients in Shenzhen.

**Methods:**

A cross-sectional study was conducted between 1 December 2024 and 31 March 2025, at Shenzhen Eye Hospital, Shenzhen People’s Hospital, and Shenzhen Nanshan People’s Hospital. Data were gathered using structured questionnaires designed to measure demographic information, clinical features, and KAP scores regarding cataracts. Additionally, potential factors affecting KAP outcomes were examined through statistical analysis.

**Results:**

Among the 500 participating cataract patients, 299 (59.8%) were female, and 185 (37.0%) had previously received cataract surgery. The mean (SD) knowledge, attitude, and practice scores were 9.22 (4.35) (possible range: 0–17), 45.85 (3.78) (possible range: 11–55), and 27.18 (2.61) (possible range: 8–40), respectively. Correlation analysis showed that there were significant positive correlations between knowledge and practice (r = 0.276, *p* < 0.001). Furthermore, there was a correlation between attitude and practice (r = 0.329, *p* < 0.001). Analysis of the direct and indirect effects of the model showed that attitudes had a direct effect on practice (*β* = 0.586, *p* < 0.001). However, neither the direct effect of knowledge on attitudes and practice nor the indirect effect of knowledge on practice was significant.

**Conclusion:**

Shenzhen cataract patients demonstrated insufficient knowledge and positive attitudes but passive practices, with attitudes directly shaping behaviors. Healthcare providers should enhance patient education and tailor communication for older populations to improve engagement and cataract management.

## Introduction

Cataract is one of the most common ophthalmic diseases worldwide and remains a leading cause of vision loss. Recent global analyses show that cataract remains the leading cause of blindness worldwide, accounting for more than 40% of all blindness cases. According to Wang et al. (1), the global burden of cataract has continued to rise over the past three decades, with increasing disability-adjusted life years (DALYs) and a growing number of people affected by cataract-related visual impairment. According to the World Health Organization, cataracts account for approximately half of blindness cases, affecting over 65.2 million people with moderate or severe distance vision impairment (2, 3). In China, from 1990 to 2019, the number of people with moderate vision impairment increased by 133.67%, severe vision impairment by 147.14%, and blindness by 64.35% (4, 5). With China’s rapidly aging population, the number of cataract cases in people aged 45–89 is expected to surpass 240.83 million by 2050, representing a prevalence rate of approximately one-third of this population (4).

Despite substantial advancements in cataract surgery, which remains the only effective treatment for cataracts, patients’ knowledge, attitudes, and practices regarding treatment still vary considerably. Surgery has been rated by the WHO as one of the most cost-effective medical procedures that improves quality of life and increases household economic productivity (6). However, China’s cataract surgery rate (CSR) remains low compared to developed countries, increasing from 370 per million population in 2000 to 2,205 in 2017, still far below countries such as France, where the CSR exceeded 10,000 in 2012 (4). The main barriers to cataract surgery uptake include cost, lack of knowledge about cataracts, and concerns about surgical quality (4). One study found that the leading obstacle was low patient awareness of cataracts as the cause of visual impairment (74.5%), followed by failure to recognize the necessity of surgery (7.4%) and financial concerns (5.5%) (7). The knowledge-attitude-practice (KAP) theory suggests that improved knowledge positively influences attitudes, which in turn shape health-seeking behaviors, with evidence showing that patients with a better understanding experience greater certainty about their treatment options (8, 9).

Previous studies on cataract awareness and treatment have primarily been conducted in rural China or in provinces with varying economic development levels. Research in western China found that more than one-third of participants had poor knowledge of cataracts, especially regarding treatment options and surgical reimbursement (8, 9). A nationally representative study demonstrated that while both rural and urban residents have access to cataract surgery, underserved groups such as rural dwellers and those with lower household incomes, are less likely to receive it (6, 10, 11). However, these findings may not be directly applicable to Shenzhen’s unique urban context with its advanced healthcare infrastructure and educated population. This study aims to bridge this research gap by conducting a comprehensive assessment of the knowledge, attitudes, and practices of cataract patients in Shenzhen regarding cataract treatment. By understanding the factors that influence treatment decisions in this specific population, including socioeconomic status, education level, and healthcare accessibility, we can provide targeted recommendations to improve early diagnosis and treatment uptake, ultimately enhancing visual health outcomes and quality of life for cataract patients in this rapidly developing urban center.

## Materials and methods

### Study design and participants

This cross-sectional study was conducted to assess patients diagnosed with cataracts at three medical institutions in Shenzhen, Shenzhen Eye Hospital, Shenzhen People’s Hospital, and Shenzhen Nanshan People’s Hospital, from 1 December 2024 to 31 March 2025. Ethical approval was granted by the Ethics Committee of Shenzhen Eye Hospital (approval number: 2024-062(Y)-02), and informed consent was obtained from all participants prior to participation. Specifically, Shenzhen Eye Hospital is a specialized tertiary ophthalmic institution that performs approximately 50% of all cataract surgeries in the city, while Shenzhen People’s Hospital and Shenzhen Nanshan People’s Hospital represent a city-level and a district-level general hospital, respectively. Together, these three hospitals account for approximately 77% of cataract surgeries performed in Shenzhen, supporting the representativeness of the study sample.

Eligible participants included patients clinically diagnosed with various forms of cataracts, including age-related cataracts, complicated cataracts, and metabolic cataracts. All participants presented distinct symptoms, primarily characterized by painless visual impairment and clinically observable lens opacification. Written informed consent was obtained from all included patients.

The inclusion criteria required participants to have a clear clinical diagnosis of cataracts accompanied by symptomatic visual deterioration. Patients were excluded if they had cognitive impairment or other conditions impeding their ability to provide reliable information.

### Procedures

The questionnaire was developed based on a comprehensive review of existing literature and expert consensus in the field of ophthalmology and public health, ensuring content relevance and clarity. Following the initial draft, the research team conducted internal reviews and iterative discussions to refine its content and structure. A small-scale pilot study was then conducted with 30 cataract patients whose demographic characteristics and clinical profiles were consistent with those of the target study population. The pilot test demonstrated strong internal consistency, with a Cronbach’s alpha coefficient of 0.848. Upon formal deployment, questionnaires were carefully checked for completeness and logical consistency immediately after data collection. Completeness verification ensured the absence of missing responses, while logical consistency checks identified and corrected inconsistencies or contradictory responses.

The questionnaire comprised four key dimensions. The first dimension gathered demographic information. The knowledge dimension included 17 single-choice questions, with each correct response scored at 1 point, while incorrect or uncertain responses received no points; scores ranged from 0 to 17, with higher scores reflecting better knowledge. The attitude dimension consisted of 11 items evaluated on a 5-point Likert scale, with scores ranging from 11 to 55; higher scores indicated more positive attitudes. This scoring approach is consistent with validated Likert-based KAP instruments reported in recent questionnaire development studies (12). The practice dimension comprised 8-point Likert scale items and 1 open-ended question (without scoring), resulting in total possible scores from 8 to 40; higher scores reflected better practical behavior regarding cataract treatment. The structure and scoring of the practice items are aligned with validated KAP questionnaires that use Likert response formats in recent methodological studies (12). Attaining scores above 70% of the maximum in each section indicated adequate knowledge, positive attitude, and proactive practice (13).

Due to the advanced age of many participants, data were primarily collected using printed paper questionnaires. These were completed in person under the supervision of trained research assistants at the participating hospitals. After completion, the responses were entered into the Questionnaire Star platform by the research assistants and underwent double-checking by two independent reviewers to ensure data accuracy and prevent input errors. This approach ensured the inclusion of older individuals who may not be familiar with smartphones or digital platforms, thus minimizing potential age-related selection bias. The questionnaire used in this study can be accessed via the following link: https://www.wjx.cn/.

### Sample size determination

The sample size estimation for this study was influenced by several considerations. Initially, previous KAP studies on cataracts provided benchmarks for anticipated rates of patient awareness, appropriate attitudes, and behavioral adherence (13). Utilizing these reference rates, the sample size was calculated using the standard formula:


$$n=\frac{z^{2}p(1-p)}{d^{2}}$$

where ‘z’ represents the standard normal deviation (z-score) at a 95% confidence interval, ‘p’ represents the expected proportion based on existing literature, and ‘d’ represents the margin of error. Consequently, the initial computed sample size was 384, assuming an expected population proportion consistent with comparable cataract-related KAP studies. Given the specialized nature and potential heterogeneity of the target population, individuals undergoing cataract surgery, an additional adjustment was made to ensure the representativeness and robustness of findings. Furthermore, to account for a potential non-response or attrition rate of approximately 10%, the final adjusted sample size was increased to 555 participants, ensuring adequate statistical power and reliability for subsequent analyses.

### Sampling method

The target population consisted specifically of individuals diagnosed with cataracts who were scheduled for or had undergone cataract surgery. To ensure representative sampling reflective of patient distribution, stratified sampling was utilized. This approach considered differing patient volumes across healthcare institutions in Shenzhen, notably differentiating between specialized ophthalmology centers and general hospitals. Consequently, Shenzhen Eye Hospital, a specialized institution with high patient volume, and two general hospitals—Shenzhen People’s Hospital (a city-level hospital) and Shenzhen Nanshan People’s Hospital (a district-level hospital)—were selected for inclusion. Together, these three hospitals accounted for approximately 77% of all cataract diagnoses and treatments conducted in Shenzhen in 2023, thereby ensuring that the sample broadly reflects the city’s cataract patient population. Participants from each facility were randomly chosen using computer-generated random numbers derived from surgical patient records within the defined study period. By including both a major specialized ophthalmology hospital and general hospitals, this sampling method facilitated comprehensive data collection across varying clinical settings, contributing to the robustness and generalizability of the study results.

### Statistical analyses

Data analysis was performed using SPSS 22.0 (IBM, Armonk, NY, United States) and R software version 4.3.2. Continuous variables were summarized as means with standard deviations (SDs) for normally distributed data and medians with interquartile ranges (25th–75th percentiles) for data that did not follow a normal distribution. Categorical variables were expressed as frequency counts and percentages (*n*, %). Normality of continuous variables was assessed using the Kolmogorov–Smirnov test. Comparisons of normally distributed continuous data between groups were conducted using independent-sample t-tests, whereas the Wilcoxon–Mann–Whitney U-test was utilized for non-normally distributed data. For comparisons involving more than two groups, analysis of variance (ANOVA) was applied when the data distribution was normal, while the Kruskal–Wallis H test was used when the data did not follow a normal distribution. Correlation analyses among KAP dimension scores were conducted using Pearson’s correlation coefficient if data met assumptions of normality; otherwise, Spearman’s rank correlation coefficient was used. Univariate and multivariate regression analyses were conducted separately for each dimension (knowledge, attitude, and practice) as dependent variables to investigate associated demographic and clinical predictors. Statistical significance was determined at a two-sided *p*-value of less than 0.05. Structural equation modeling (SEM) was further conducted based on the knowledge-attitude-practice theoretical framework to explore whether attitude mediates the association between knowledge and practice. Both direct and indirect effects were quantified and compared. The goodness-of-fit for the SEM was evaluated using the root mean square error of approximation (RMSEA), incremental fit index (IFI), Tucker–Lewis index (TLI), and comparative fit index (CFI). Indirect mediation effects and direct relationships among variables were also calculated and interpreted to elucidate the underlying mechanisms connecting knowledge, attitudes, and practices.

## Results

In the formal experiment, the overall Cronbach’s *α* coefficient and subscale internal consistency were good, with a total Cronbach’s α of 0.8445, and the KMO value of the overall scale was 0.9057 (Supplementary Table S6).

### Demographic information on participants

Among the 500 cataract patients who participated in this study, 299 (59.8%) were female, 205 (41.0%) were aged 65–74 years, 318 (63.6%) had other chronic diseases, 360 (72.0%) had family members with cataracts, 383 (76.6%) were government employees/public institution staff, and 185 (37.0%) had undergone cataract surgical treatment. The mean (SD) knowledge, attitude, and practice scores were 9.22 (4.35) (possible range: 0–17), 45.85 (3.78) (possible range: 11–55), and 27.18 (2.61) (possible range: 8–40), respectively. Analyses of demographic characteristics found that participants’ knowledge, attitude, and practice scores varied across highest education (*p* = 0.008, *p* = 0.047, *p* = 0.001), monthly income per capita (*p* = 0.016, *p* < 0.001, *p* = 0.006), classification of the hospital (*p* < 0.001, *p* = 0.021, *p* < 0.001), and duration of medical consultation (*p* = 0.010, *p* = 0.004, *p* = 0.021). Meanwhile, knowledge scores varied significantly by the presence of a family member with cataracts (*p* < 0.001), cataract surgery status (*p* < 0.001), and source of information on cataract treatment (*p* < 0.001). Attitude scores varied significantly by age (*p* = 0.010), presence of a family member with cataracts (*p* < 0.001), and occupation (*p* = 0.003). Practice scores varied significantly by medical insurance (*p* = 0.002), cataract surgery status (*p* < 0.001), and source of information about cataract treatment (*p* < 0.001) (Table 1).

**Table 1:** Baseline characteristics.

*N* = 500	*N* (%)	Knowledge	*P*	Attitude	*P*	Practice	*P*
		Mean (SD)		Mean (SD)		Mean (SD)	
Total score	500 (100.0)	9.22 (4.35)		45.85 (3.78)		27.18 (2.61)	
Gender			0.726		0.242		0.541
Male	201 (40.2)	9.25 (4.30)		45.60 (3.67)		27.22 (2.51)	
Female	299 (59.8)	9.19 (4.38)		46.01 (3.84)		27.16 (2.67)	
Age			0.517		**0.010**		0.594
Under 65	102 (20.4)	9.59 (4.05)		46.45 (4.06)		27.38 (2.53)	
65–74	205 (41.0)	9.17 (4.19)		46.01 (3.84)		27.24 (2.77)	
75 or more	193 (38.6)	9.07 (4.66)		45.35 (3.50)		27.01 (2.46)	
Highest education			**0.008**		**0.047**		**0.001**
Bachelor’s degree or above	82 (16.4)	9.66 (4.22)		47.15 (4.43)		27.68 (2.43)	
Primary school or below	141 (28.2)	8.77 (4.51)		45.65 (3.52)		26.65 (2.44)	
Junior high school	82 (16.4)	8.05 (4.24)		46.01 (3.79)		26.91 (3.08)	
Senior high school/technical secondary school	61 (12.2)	9.11 (4.52)		45.77 (4.16)		27.03 (3.11)	
Associate degree	134 (26.8)	10.17 (4.04)		45.19 (3.23)		27.66 (2.17)	
Other chronic diseases			0.826		0.968		0.796
No	182 (36.4)	9.15 (4.31)		45.91 (3.86)		27.14 (2.79)	
Yes	318 (63.6)	9.25 (4.37)		45.81 (3.73)		27.20 (2.50)	
Hypertension	219 (43.8)	8.99 (4.48)		45.75 (3.72)		27.02 (2.50)	
Diabetes	106 (21.2)	9.23 (4.29)		45.69 (3.86)		27.25 (2.60)	
Heart disease	77 (15.4)	9.84 (4.17)		46.47 (4.22)		27.47 (2.34)	
Glaucoma	2 (0.4)	13.50 (2.12)		44.00 (0.00)		31.00 (2.83)	
Other	80 (16.0)	9.45 (4.27)		45.65 (3.32)		27.21 (2.46)	
Family member with cataracts			**<0.001**		**<0.001**		0.326
Yes	360 (72.0)	9.61 (4.32)		45.46 (3.51)		27.23 (2.43)	
No	140 (28.0)	8.21 (4.27)		46.85 (4.25)		27.06 (3.01)	
Monthly income per capita			**0.016**		**<0.001**		**0.006**
Below 7,000	151 (30.2)	9.28 (4.33)		45.11 (3.18)		26.79 (2.55)	
7,000–9,000	95 (19.0)	9.44 (4.42)		45.57 (3.63)		27.13 (2.16)	
9,000–11,000	125 (25.0)	9.92 (4.18)		45.77 (3.87)		27.11 (2.25)	
Above 11,000	129 (25.8)	8.29 (4.36)		46.98 (4.20)		27.75 (3.16)	
Medical insurance			0.100		0.902		**0.002**
Shenzhen medical insurance	251 (50.2)	9.55 (4.33)		45.88 (3.78)		27.57 (2.61)	
Non-local medical insurance	236 (47.2)	8.96 (4.28)		45.85 (3.83)		26.80 (2.56)	
Other	13 (2.6)	7.46 (5.36)		45.08 (2.63)		26.62 (2.36)	
Occupation			0.623		**0.003**		0.264
Medical educators	36 (7.2)	8.92 (3.96)		44.53 (2.58)		26.75 (2.66)	
Government employee/public institution staff	383 (76.6)	9.32 (4.40)		45.78 (3.79)		27.19 (2.54)	
Business owner	60 (12.0)	8.93 (4.38)		46.28 (3.61)		27.47 (2.63)	
Other	21 (4.2)	8.67 (4.15)		48.00 (4.85)		26.95 (3.58)	
Classification of the hospital			**<0.001**		**0.021**		**<0.001**
Tertiary A (Grade 3A)	456 (91.2)	9.49 (4.26)		45.78 (3.73)		27.31 (2.38)	
Tertiary (Grade 3)	14 (2.8)	8.64 (4.13)		47.93 (4.30)		28.07 (2.87)	
Not sure	30 (6.0)	5.33 (3.99)		45.93 (4.10)		24.83 (4.23)	
Duration from symptom detection to medical consultation			**0.010**		**0.004**		**0.024**
Within 6 months	37 (7.4)	7.65 (4.03)		46.73 (4.10)		26.24 (2.84)	
6 months to 1 year	37 (7.4)	8.16 (4.58)		46.84 (3.82)		26.43 (2.92)	
More than 1 year	426 (85.2)	9.44 (4.32)		45.68 (3.73)		27.33 (2.53)	
Cataract surgery			**<0.001**		0.73		**<0.001**
Yes	185 (37.0)	13.36 (2.49)		45.85 (3.89)		27.92 (2.59)	
No	315 (63.0)	6.78 (3.21)		45.84 (3.72)		26.75 (2.52)	
Source of information on cataract treatment			**<0.001**		0.489		**<0.001**
Consultation with doctors	293 (58.6)	11.29 (3.84)		45.84 (3.84)		27.69 (2.32)	
New media or online searches, Recommendations from family and friends	207 (41.4)	6.28 (3.16)		45.86 (3.70)		26.46 (2.81)	

Bold values indicate statistical significance (*p* < 0.05).

### Knowledge, attitude, and practice

The distribution of knowledge dimensions showed that the three questions with the highest number of participants choosing the “Not sure” option were “Secondary glaucoma due to cataracts usually occurs during the immature/swollen stage and the hypermature stage” (K2) with 67.2%, “Common complications of cataract surgery include ptosis (drooping eyelid), dry eye, and decreased vision” (K5) with 65%, and “Cataract surgery combined with goniosynechialysis can simultaneously address cataracts and intraocular pressure issues” (K6) with 63.4% (Supplementary Table S1).

Responses to the attitude dimension showed that 19% strongly agreed and 79.8% agreed that cataracts significantly impact daily life (A1), and 20.4% strongly agreed and 78.2% agreed that the government or medical institutions should strengthen cataract treatment awareness and promotion (A8) (Supplementary Table S2).

Responses to the practice dimension showed that 15.6% rarely and 74.6% never feel the improvement of cataract treatment using eye drops (P4e), 72.8% rarely and 6% never participate in cataract health education activities organized by community or medical institutions (P5), and 39.8% rarely and 2.4% never undergo eye health check-ups (P2) (Supplementary Table S3).

### Correlations between KAP

Correlation analysis showed that there were significant positive correlations between knowledge and practice (r = 0.276, *p* < 0.001). Furthermore, there was a correlation between attitude and practice (r = 0.329, *p* < 0.001) (Table 2).

**Table 2:** Correlation analysis.

Spearman	Knowledge	Attitude	Practice
Knowledge	1.000		
Attitude	−0.072 (*p* = 0.110)	1.000	
Practice	0.276 (*p* < 0.001)	0.329 (*p* < 0.001)	1.000

### Factors associated with KAP

The median of the knowledge, attitude, and practice total scores was used as the cutoff value for each dimension to divide the groups, and the number of participants above the cutoff value was 275 (55%), 442 (88.4%), and 297 (59.4%), respectively. Multivariate logistic regression showed that not being sure about the classification of the hospital (OR = 0.275, 95% CI: [0.086, 0.884], *p* = 0.030), not having cataract surgery (OR = 0.033, 95% CI: [0.015, 0.073], *p* < 0.001), and getting information from new media, online searches, and recommendations from family and friends (OR = 0.292, 95% CI: [0.173, 0.493], *p* < 0.001) were independently associated with poor knowledge. Concurrently, being 75 years or older (OR = 0.214, 95% CI: [0.074, 0.623], *p* = 0.005), being a government employee/public institution staff member (OR = 3.398, 95% CI: [1.309, 8.822], *p* = 0.012), and being a business owner (OR = 5.422, 95% CI: [1.559,18.860], *p* = 0.008) were independently associated with attitude. Furthermore, knowledge (OR = 1.117, 95% CI: [1.035, 1.206], *p* = 0.005), attitude (OR = 1.170, 95% CI: [1.096, 1.248], *p* < 0.001), being male (OR = 0.357, 95% CI: [0.136–0.943], *p* = 0.038), having an associate degree (OR = 2.162, 95% CI: [1.114, 4.193], *p* = 0.023), being a business owner (OR = 2.965, 95% CI: [1.138, 7.723], *p* = 0.026), and more than 1 year from symptom discovery to medical consultation (OR = 2.157, 95% CI: [1.003, 4.639], *p* = 0.049) were independently associated with practice (Table 3).

**Table 3:** Univariate and multivariate analyses.

Cutoff value: median	*N* (%)
Knowledge total score	
K sum> = 10	275 (55%)
K sum<=9	225 (45%)
Attitude total score	
A sum> = 32	442 (88.4%)
A sum<=31	58 (11.6%)
Practice total score	
P sum> = 35	297 (59.4%)
P sum<=34	203 (40.6%)

Knowledge	Univariate analysis		Multivariate analysis	
	OR (95%CI)	*p*	OR (95%CI)	*p*
Gender				
Male				
Female	0.890 (0.621, 1.275)	0.527		
Age				
Under 65				
65–74	0.795 (0.490, 1.282)	0.349		
75 or more	0.853 (0.523, 1.384)	0.521		
Highest education				
Bachelor’s degree or above				
Primary school or below	0.668 (0.382, 1.157)	0.153	0.732 (0.337, 1.588)	0.430
Junior high school	0.553 (0.295, 1.025)	0.061	0.840 (0.369, 1.914)	0.678
Senior high school/technical secondary school	1.057 (0.535, 2.103)	0.873	1.108 (0.463, 2.655)	0.818
Associate degree	0.865 (0.491, 1.511)	0.611	0.721 (0.346, 1.500)	0.381
Family member with cataracts				
Yes				
No	0.671 (0.453, 0.992)	0.046	1.190 (0.687, 2.059)	0.535
Monthly income per capita				
Below 7,000				
7,000–9,000	0.991 (0.592, 1.662)	0.972		
9,000–11,000	1.457 (0.898, 2.376)	0.129		
Above 11,000	0.712 (0.444, 1.141)	0.159		
Medical insurance				
Shenzhen medical insurance				
Non-local medical insurance	0.783 (0.547, 1.119)	0.180		
Other	0.616 (0.193, 1.907)	0.397		
Occupation				
Medical educators				
Government employee/public institution staff	1.253 (0.629, 2.495)	0.518		
Business owner	1.308 (0.570, 3.014)	0.526		
Other	0.909 (0.306, 2.681)	0.862		
Classification of the hospital				
Tertiary A (Grade 3A)				
Tertiary (Grade 3)	1.005 (0.344, 3.097)	0.993	0.429 (0.071, 2.593)	0.356
Not sure	0.229 (0.090, 0.520)	0.001	0.275 (0.086, 0.884)	0.030
Duration from symptom detection to medical consultation				
Within 6 months				
6 months to 1 year	1.749 (0.692, 4.518)	0.240	2.584 (0.749, 8.919)	0.133
More than 1 year	2.475 (1.246, 5.131)	0.011	1.891 (0.761, 4.698)	0.170
Cataract surgery				
Yes				
No	0.020 (0.009, 0.041)	<0.001	0.033 (0.015, 0.073)	<0.001
Source of information on cataract treatment				
Consultation with doctors				
New media or online searches, recommendations from family and friends	0.096 (0.063, 0.145)	<0.001	0.292 (0.173, 0.493)	<0.001

Attitude	Univariate analysis		Multivariate analysis	
	OR (95%CI)	*p*	OR (95%CI)	*p*
Knowledge	1.019 (0.957, 1.086)	0.551	1.019 (0.955, 1.088)	0.562
Gender				
Male				
Female	1.057 (0.600, 1.835)	0.846		
Age				
Under 65				
65–74	0.495 (0.178, 1.184)	0.139	0.354 (0.122, 1.027)	0.056
75 or more	0.353 (0.129, 0.827)	0.026	0.214 (0.074, 0.623)	0.005
Highest education				
Bachelor’s degree or above				
Primary school or below	0.507 (0.178, 1.259)	0.166		
Junior high school	0.461 (0.153, 1.253)	0.141		
Senior high school/technical secondary school	0.609 (0.187, 1.931)	0.396		
Associate degree	0.677 (0.231, 1.766)	0.443		
Family member with cataracts				
Yes				
No	1.253 (0.679, 2.445)	0.487		
Monthly income per capita				
Below 7,000				
7,000–9,000	0.987 (0.482, 2.078)	0.971	0.855 (0.387, 1.888)	0.698
9,000–11,000	1.352 (0.667, 2.824)	0.409	1.050 (0.469, 2.349)	0.906
Above 11,000	2.580 (1.148, 6.375)	0.028	2.454 (0.990, 6.084)	0.053
Medical insurance				
Shenzhen medical insurance				
Non-local medical insurance	0.737 (0.422, 1.276)	0.277		
Other	/	/		
Occupation				
Medical educators				
Government employee/public institution staff	2.781 (1.167, 6.133)	0.015	3.398 (1.309, 8.822)	0.012
Business owner	3.667 (1.153, 12.944)	0.032	5.422 (1.559, 18.860)	0.008
Other	2.000 (0.515, 9.940)	0.344	0.718 (0.137, 3.756)	0.695
Classification of the hospital				
Tertiary A (Grade 3A)				
Tertiary (Grade 3)	/	/		
Not sure	0.515 (0.213, 1.441)	0.166		
Duration from symptom detection to medical consultation				
Within 6 months				
6 months to 1 year	1.544 (0.242, 12.276)	0.645		
More than 1 year	0.621 (0.146, 1.807)	0.442		
Cataract surgery				
Yes				
No	0.884 (0.489, 1.554)	0.673		
Source of information on cataract treatment				
Consultation with doctors				
New media or online searches, recommendations from family and friends	0.854 (0.493, 1.491)	0.573		

Practice	Univariate analysis		Multivariate analysis	
	OR (95%CI)	*p*	OR (95%CI)	*p*
Knowledge	1.124 (1.076, 1.174)	<0.001	1.117 (1.035, 1.206)	0.005
Attitude	1.125 (1.065, 1.188)	<0.001	1.170 (1.096, 1.248)	<0.001
Gender				
Male				
Female	1.014 (0.704, 1.458)	0.942		
Age				
Under 65				
65–74	1.008 (0.620, 1.632)	0.973		
75 or more	1.055 (0.645, 1.717)	0.831		
Highest education				
Bachelor’s degree or above				
Primary school or below	0.602 (0.342, 1.047)	0.075	0.831 (0.392, 1.762)	0.629
Junior high school	0.550 (0.293, 1.022)	0.060	1.017 (0.464, 2.230)	0.967
Senior high school/technical secondary school	0.777 (0.393, 1.532)	0.465	1.220 (0.548, 2.715)	0.626
Associate degree	1.571 (0.869, 2.835)	0.134	2.162 (1.114, 4.193)	0.023
Family member with cataracts				
Yes				
No	0.662 (0.446, 0.982)	0.040	0.776 (0.483, 1.249)	0.296
Monthly income per capita				
Below 7,000				
7,000–9,000	1.648 (0.978, 2.801)	0.062	1.244 (0.649, 2.381)	0.511
9,000–11,000	1.349 (0.838, 2.181)	0.219	0.677 (0.335, 1.366)	0.276
Above 11,000	1.991 (1.227, 3.257)	0.006	1.538 (0.736, 3.212)	0.252
Medical insurance				
Shenzhen medical insurance				
Non-local medical insurance	0.607 (0.421, 0.874)	0.007	0.748 (0.427, 1.310)	0.310
Other	0.326 (0.096, 1.006)	0.055	0.276 (0.066, 1.145)	0.076
Occupation				
Medical educators				
Government employee/public institution staff	2.199 (1.105, 4.475)	0.026	1.266 (0.547, 2.931)	0.582
Business owner	2.600 (1.123, 6.178)	0.027	2.965 (1.138, 7.723)	0.026
Other	1.050 (0.348, 3.125)	0.930	0.448 (0.122, 1.641)	0.226
Classification of the hospital				
Tertiary A (Grade 3A)				
Tertiary (Grade 3)	1.131 (0.384, 3.733)	0.827	1.363 (0.373, 4.978)	0.640
Not sure	0.229 (0.094, 0.505)	0.001	0.377 (0.137, 1.036)	0.058
Duration from symptom detection to medical consultation				
Within 6 months				
6 months to 1 year	1.385 (0.555, 3.496)	0.485	1.513 (0.517, 4.425)	0.450
More than 1 year	2.097 (1.067, 4.194)	0.033	2.157 (1.003, 4.639)	0.049
Cataract surgery				
Yes				
No	0.535 (0.364, 0.782)	0.001	1.491 (0.765, 2.908)	0.241
Source of information on cataract treatment				
Consultation with doctors				
New media or online searches, recommendations from family and friends	0.410 (0.283, 0.591)	<0.001	0.603 (0.359, 1.013)	0.056

### SEM analysis

**Figure 1:**
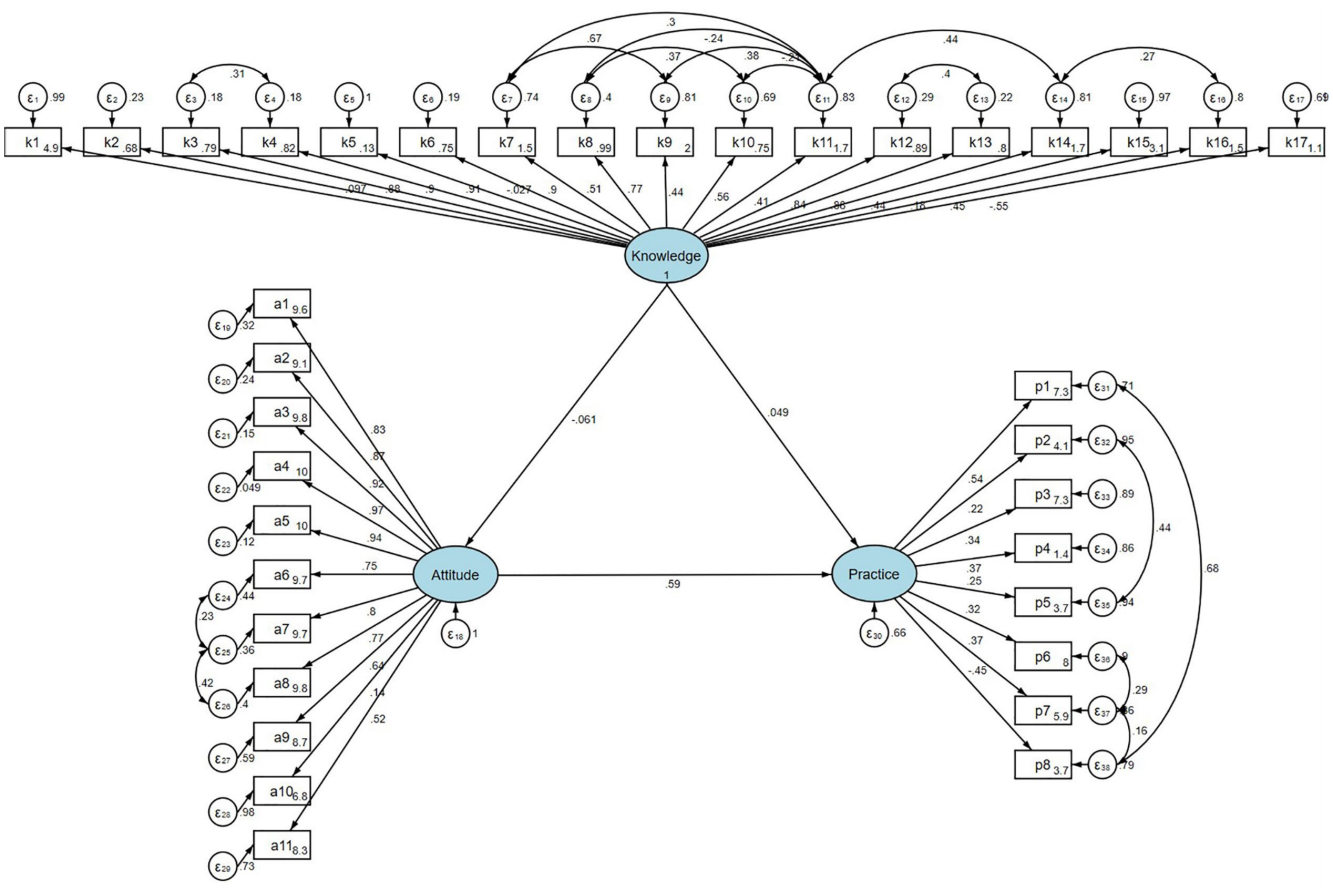
SEM model.

The fit of the SEM model yielded good indices demonstrating good model fit (RMSEA value: 0.076, SRMR value: 0.090, TLI value: 0.855, and CFI value: 0.867) (Supplementary Table S4), and the effect estimates between KAP are detailed in Supplementary Table S5. Analysis of the direct and indirect effects of the model showed that attitude had a direct effect on practice (*β* = 0.586, *p* < 0.001). However, neither the direct effect of knowledge on attitudes and practice nor the indirect effect of knowledge on practice was significant (Table 4; Figure 1).

**Table 4:** Analysis of direct and indirect effects.

Model paths		Total effects		Direct effect		Indirect effect	
		β(95%CI)	*P*	β(95%CI)	*P*	β(95%CI)	*P*
Attitude	Knowledge	−0.061 (−0.151, 0.030)	0.189	−0.061 (−0.151, 0.030)	0.189		
Practice	Knowledge	0.013 (−0.100, 0.126)	0.815	0.049 (−0.052, 0.150)	0.340	−0.036 (−0.089, 0.018)	0.192
	Attitude	0.586 (0.503, 0.669)	<0.001	0.586 (0.503, 0.669)	<0.001		

## Discussion

The study indicates that cataract patients in Shenzhen have limited knowledge and generally positive attitudes but insufficient proactive behaviors toward cataract treatment, with attitudes exerting a significant influence on their actual practices. These findings reflect a clear gap between understanding and behavior, suggesting that targeted educational and attitude-focused interventions may help strengthen patients’ engagement in cataract management.

Overall, the results highlight several important themes regarding the current KAP status of cataract patients in an urban setting. Consistent with previous studies on chronic diseases and eye care management, the mismatch between knowledge, attitudes, and practices has also been observed elsewhere (14, 15). Prior research conducted in both developed and less developed regions of China has documented disparities in cataract surgery uptake, health literacy, and access to ophthalmic information, often driven by inconsistent health education or variable availability of clinical guidance. Despite Shenzhen’s strong medical infrastructure, the mean knowledge score in this study (9.22 out of 17) was only slightly higher than that reported in studies from less urbanized regions, indicating that healthcare resource availability alone does not ensure adequate patient education. This underscores an important insight: Urbanization does not automatically translate into improved awareness of eye health.

The detailed knowledge assessment further revealed substantial gaps in patients’ understanding of surgical complications, treatment options, and postoperative expectations. This pattern mirrors findings from studies conducted in other regions, which similarly reported limited comprehension of ophthalmic conditions and treatment processes (16, 17). Although knowledge levels in this study surpassed those reported in rural populations—where accuracy rates are often below 30% (18)—they remained insufficient for informed decision-making. Comparisons with research from Beijing and Shanghai, where structured ophthalmic education is more widely implemented, further illustrate that even well-resourced cities such as Shenzhen face persistent challenges in delivering consistent and comprehensible eye health information.

A notable finding of this study is the disconnect between overwhelmingly positive attitudes and relatively inactive practical behaviors. In well-developed urban contexts, individuals often express supportive attitudes toward medical care due to broader exposure to health messaging, yet this does not always translate into action. Our SEM results showed that attitude—not knowledge—had a direct effect on practice, diverging from the classic KAP model. This pattern is consistent with observations in chronic disease management, where emotional readiness, communication quality, and perceived relevance often exert stronger behavioral influence than factual knowledge (19). The present study extends these insights to ophthalmic care and highlights the importance of prioritizing motivational and communication factors in future interventions.

The study also revealed meaningful variations across demographic subgroups. Older patients, especially those aged 75 years or older, displayed less positive attitudes, a trend also reported in studies involving older adults who tend to have heightened concerns about surgical risks or limited confidence in navigating medical systems (19–21). Moreover, patients who rely primarily on new media or informal recommendations demonstrated significantly poorer knowledge than those who consulted physicians. This finding reflects growing concerns about the accuracy and reliability of online health information and emphasizes the need for standardized, professionally supervised educational content.

These findings offer several implications for improving cataract care in urban public health systems. First, structured educational programs delivered through community health centers and hospital outpatient departments may help correct misconceptions and enhance understanding of cataract surgery. Second, authoritative digital resources—such as official new-media platforms operated by medical institutions—could be used to disseminate clear, standardized cataract education messages, reducing the influence of unreliable online content. Third, the pronounced KAP discrepancies in patients aged ≥75 years highlight the need for age-tailored communication strategies that incorporate simplified explanations, slower-paced discussions, and more personalized counseling. Fourth, integrating cataract screening and education into routine medical insurance workflows or chronic disease follow-up systems could improve treatment conversion rates by linking eye care to existing patient management frameworks. These strategies reflect an approach increasingly recommended in integrated chronic disease programs and may help address both behavioral and structural barriers to care.

Examination of the distribution revealed that routine eye check-ups and community-based cataract education activities were particularly underutilized, indicating potential gaps in the healthcare system’s preventive service provision and public outreach effectiveness. In Shenzhen, an integrated “medical-prevention collaboration” system has been implemented to strengthen public health services, including regular ophthalmology outreach visits by hospital-based specialists to community health centers. However, our findings suggest that ophthalmic health education has not been effectively aligned with this system. Future health promotion efforts should leverage the existing medical-prevention framework to enhance the accessibility and continuity of cataract-related education and screening, particularly for older adults in the community.

This mirrors broader regional healthcare delivery patterns where preventive eye care services and structured community-based education remain under-resourced or inconsistently implemented (18, 22). Strategic adjustments at the healthcare-system level, such as expanding community outreach programs and enhancing the role of primary care settings in ophthalmologic care, could significantly bridge these gaps. Training primary care providers to proactively identify cataract-related symptoms and provide initial counseling could also foster more consistent patient engagement. Educational strategies should be stratified based on patients’ information-seeking behaviors; for instance, those who rely primarily on physician consultations may benefit from enhanced face-to-face explanations during clinic visits, while patients obtaining information from new media may require structured digital content delivered through authoritative platforms. Additionally, policy-level integration between health education and medical insurance reimbursement mechanisms—for example, linking cataract-related health education participation to enhanced reimbursement eligibility—may further incentivize proactive engagement and strengthen health literacy.

This study has several limitations. First, the cross-sectional design restricts the ability to establish causality or observe changes in KAP over time. Second, self-reported questionnaires might introduce response bias, including recall bias or social desirability bias, affecting data accuracy. Third, the generalizability of the findings may be limited, as participants were recruited only from three selected hospitals in Shenzhen. However, these included one specialized ophthalmology hospital, one city-level general hospital, and one district-level hospital, collectively accounting for approximately 77% of the city’s cataract surgeries. This enhances the representativeness of the sample, although patients outside the hospital-based system or those seeking care in private or smaller community clinics may still be underrepresented.

In conclusion, this study demonstrates that cataract patients in Shenzhen—a highly urbanized city with relatively advanced healthcare resources—still possess insufficient knowledge despite maintaining generally favorable attitudes. Their practical engagement in cataract care remains inadequate, with attitudes exerting a significant influence on their behavior. These findings highlight that urbanization alone does not guarantee adequate health literacy or proactive health behavior. To address these gaps, clinicians and policymakers should design stratified educational interventions tailored to different information-seeking behaviors and fully leverage existing “medical-prevention integration” programs. Integrating ophthalmic health education into community-based services and aligning such efforts with broader healthcare delivery and insurance frameworks may offer an effective path to improving cataract awareness and management outcomes in urban populations. In terms of public health implications, the findings highlight several priorities for improving cataract management in urban populations. Strengthening structured community and clinic-based education programs, standardizing cataract-related information through official digital platforms, and integrating eye health promotion into routine medical insurance and chronic-disease follow-up systems may help increase early diagnosis and treatment uptake. The pronounced KAP gaps observed among adults aged ≥75 years suggest the need for age-tailored communication strategies that provide clearer explanations and more personalized counseling. Future research could further explore longitudinal behavioral changes and evaluate targeted interventions designed to bridge the attitude–practice gap identified in this study.

## Data Availability

The original contributions presented in the study are included in the article/Supplementary material, further inquiries can be directed to the corresponding author.
