# Supplementary material for: Knowledge, attitudes, and practices of cataract patients in Shenzhen regarding cataract treatment
Source: Front Public Health. 2026 Jan 21;13:1697694. doi: 10.3389/fpubh.2025.1697694 (PMC12867828; doi:10.3389/fpubh.2025.1697694)
Supplement: Supplementary file 1 [file Table_1.docx]

**Supplementary table 1. Distribution o f knowledge dimension responses**

| **Knowledge** | Option a | Option b | Option c | Option d | Option e |
| --- | --- | --- | --- | --- | --- |
|  | Medication | Surgery | Wearing glasses | Laser treatment | Not sure |
| 1.Which of the following is the best treatment option for cataracts? | 2 (0.4%) | 480 (96%) | 3 (0.6%) | 1 (0.2%) | 14 (2.8%) |
|  | Correct | Incorrect | Not sure |  |  |
| 2.Secondary glaucoma due to cataracts usually occurs during the immature/swollen stage and the hypermature stage | 159 (31.8%) | 5 (1%) | 336 (67.2%) |  |  |
| 3.If cataracts are not surgically treated and the lens is removed, it may cause inflammation or secondary glaucoma | 192 (38.4%) | 2 (0.4%) | 306 (61.2%) |  |  |
| 4.The key focus of treating cataracts with glaucoma is controlling intraocular pressure and protecting the optic nerve, rather than primarily improving vision | 202 (40.4%) | 2 (0.4%) | 296 (59.2%) |  |  |
| 5.Common complications of cataract surgery include ptosis (drooping eyelid), dry eye, and decreased vision | 167 (33.4%) | 8 (1.6%) | 325 (65%) |  |  |
| 6.Cataract surgery combined with goniosynechialysis can simultaneously address cataracts and intraocular pressure issues | 181 (36.2%) | 2 (0.4%) | 317 (63.4%) |  |  |
| 7.The selection of an appropriate intraocular lens for cataract surgery should be based on individual differences and the doctor's recommendations. | 352 (70.4%) | 4 (0.8%) | 144 (28.8%) |  |  |
| 8.Cataracts may progress more rapidly in individuals with certain conditions, such as diabetes, hypertension, or obesity | 248 (49.6%) | 8 (1.6%) | 244 (48.8%) |  |  |
| 9.It is recommended to consider cataract surgery when cataracts begin to affect your quality of life or hinder your ability to perform daily activities, such as reading at night or driving | 397 (79.4%) | 0 (0%) | 103 (20.6%) |  |  |
| 10.Some patients may be unable to use an intraocular lens due to other eye conditions. In such cases, vision correction can be achieved by wearing glasses or contact lenses after cataract removal. | 181 (36.2%) | 3 (0.6%) | 316 (63.2%) |  |  |
| 11.If both eyes require cataract surgery, the second eye is usually operated on only after the first eye has healed | 358 (71.6%) | 17 (3.4%) | 125 (25%) |  |  |
| 12.Cataract surgery can be performed even when there are concurrent retinal diseases | 221 (44.2%) | 5 (1%) | 274 (54.8%) |  |  |
| 13.Cataract-induced glaucoma or the risk of its onset is an indication for cataract surgery | 194 (38.8%) | 1 (0.2%) | 305 (61%) |  |  |
| 14.Most patients experience significant vision improvement within a month after cataract surgery. Vision should stabilize, and no major discomfort should persist | 370 (74%) | 0 (0%) | 130 (26%) |  |  |
|  | One week | Two weeks | Three weeks | Four weeks |  |
| 15.How long is postoperative medication typically required after cataract surgery? | 9 (1.8%) | 34 (6.8%) | 5 (1%) | 452 (90.4%) |  |
|  | Correct | Incorrect | Not sure |  |  |
| 16.After applying eye drops, pressing gently on the inner corner of the eye for one minute can prevent the medication from draining into the tear ducts and reduce systemic absorption | 340 (68%) | 22 (4.4%) | 138 (27.6%) |  |  |
| 17.Lutein can be used to treat cataracts | 20 (4%) | 207 (41.4%) | 273 (54.6%) |  |  |

**Supplementary table 2. Distribution of attitude dimension responses**

| **Attitude** | Strongly agree | Agree | Neutral | Disagree | Strongly disagree |
| --- | --- | --- | --- | --- | --- |
| 1.Do you think cataracts significantly impact daily life? | 95 (19%) | 399 (79.8%) | 5 (1%) | 0 (0%) | 1 (0.2%) |
| 2.When you first learned that you had cataracts, were you willing to actively seek treatment? | 103 (20.6%) | 383 (76.6%) | 13 (2.6%) | 1 (0.2%) | 0 (0%) |
| 3.Are you willing to undergo cataract surgery? | 94 (18.8%) | 396 (79.2%) | 10 (2%) | 0 (0%) | 0 (0%) |
| 4.Are you willing to actively seek medical help and attend regular follow-ups? | 95 (19%) | 403 (80.6%) | 2 (0.4%) | 0 (0%) | 0 (0%) |
| 5.Are you willing to communicate with your doctor and actively address any discomfort or issues during treatment? | 98 (19.6%) | 400 (80%) | 2 (0.4%) | 0 (0%) | 0 (0%) |
| 6.Are you willing to participate in cataract treatment awareness and educational activities? | 74 (14.8%) | 407 (81.4%) | 18 (3.6%) | 1 (0.2%) | 0 (0%) |
| 7.Do you think there are sufficient cataract diagnosis and treatment resources in Shenzhen? | 106 (21.2%) | 389 (77.8%) | 4 (0.8%) | 1 (0.2%) | 0 (0%) |
| 8.Do you think the government or medical institutions should strengthen cataract treatment awareness and promotion? | 102 (20.4%) | 391 (78.2%) | 7 (1.4%) | 0 (0%) | 0 (0%) |
| 9.Are you satisfied with your previous cataract treatment plan? | 112 (22.4%) | 380 (76%) | 6 (1.2%) | 0 (0%) | 2 (0.4%) |
| 10.Are you concerned about the cost of cataract surgery? | 4 (0.8%) | 9 (1.8%) | 23 (4.6%) | 381 (76.2%) | 83 (16.6%) |
| 11.Do you believe cataract surgery plays an important role in improving vision and quality of life? | 113 (22.6%) | 377 (75.4%) | 4 (0.8%) | 5 (1%) | 1 (0.2%) |

**Supplementary table 3. Distribution of practice dimension responses**

| **Practice** | Frequently | Regularly | Occasionally | Rarely | Never |
| --- | --- | --- | --- | --- | --- |
| 1.Do you pay attention to eye health protection in daily life? | 42 (8.4%) | 397 (79.4%) | 50 (10%) | 9 (1.8%) | 2 (0.4%) |
| 2.How often do you undergo eye health check-ups? | 1 (0.2%) | 30 (6%) | 258 (51.6%) | 199 (39.8%) | 12 (2.4%) |
| 3.Do you follow medical advice for medication treatment? | 108 (21.6%) | 371 (74.2%) | 8 (1.6%) | 12 (2.4%) | 1 (0.2%) |
| 4.What methods have you used to treat cataracts? (multiple choice) | Choose this | Not choose this |  |  |  |
| 4 (a.Wearing glasses) | 12 (2.4%) | 488 (97.6%) |  |  |  |
| 4 (b.Surgery) | 406 (81.2%) | 94 (18.8%) |  |  |  |
| 4 (c.Laser treatment ) | 1 (0.2%) | 499 (99.8%) |  |  |  |
| 4 (d.Medication) | 263 (52.6%) | 237 (47.4%) |  |  |  |
| 4 (e.After using eye drops for cataract treatment, did you notice an improvement?) | 36 (7.2%) | 6 (1.2%) | 7 (1.4%) | 78 (15.6%) | 373 (74.6%) |
| 5.How often do you participate in cataract health education activities organized by community or medical institutions? | 3 (0.6%) | 11 (2.2%) | 92 (18.4%) | 364 (72.8%) | 30 (6%) |
| 6.During treatment, have you experienced pain, discomfort, or other adverse reactions? | 1 (0.2%) | 1 (0.2%) | 8 (1.6%) | 214 (42.8%) | 276 (55.2%) |
| 7.After undergoing cataract treatment, do you feel your vision has improved? | 209 (41.8%) | 257 (51.4%) | 20 (4%) | 9 (1.8%) | 5 (1%) |
| 8.Do you protect your eyes from sunlight in daily life? | 184 (36.8%) | 188 (37.6%) | 52 (10.4%) | 71 (14.2%) | 5 (1%) |

**Supplementary table4. SEM fit indicators**

| **Indicators** | **Reference** | **Results** |
| --- | --- | --- |
| RMSEA | <0.08 | 0.076 |
| SRMR | <0.08 | 0.090 |
| TLI | >0.80 | 0.855 |
| CFI | >0.80 | 0.867 |

**Supplementary table 5. SEM effect estimates**

| **Indicators** |  | **Estimate** | **P>\|z\|** |
| --- | --- | --- | --- |
| Attitude |  |  |  |
|  | Knowledge | -1.31 | 0.189 |
| Practice |  |  |  |
|  | Knowledge | 0.23 | 0.815 |
|  | Attitude | 13.84 | <0.001 |

**Supplementary table 6. Cronbach’s alpha coefficients for reliability testing**

| **Analysis Index** | **Pilot Test (n = 30)** | **Formal Survey (n = 500)** |
| --- | --- | --- |
| **Cronbach’s α (Overall)** | 0.848 | 0.8445 |
| **KMO Value** | – | 0.9057 |
